# Surgical treatment of scoliosis in Smith-Magenis syndrome: a case report

**DOI:** 10.1186/1752-1947-4-26

**Published:** 2010-01-28

**Authors:** Athanasios I Tsirikos, Alexander DL Baker, Claire McClean

**Affiliations:** 1 Scottish National Spine Deformity Centre, Royal Hospital for Sick Children, Sciennes Road, Edinburgh, EH9 1LF, UK

## Abstract

**Introduction:**

Smith-Magenis syndrome is a rare genetic condition associated with scoliosis in approximately 30% of cases. There is limited information in the literature on the treatment of scoliosis and the surgical outcome in patients with this condition. Characteristic features of the syndrome, such as the presence of congenital heart and renal disease, inherent immunodeficiency, as well as severe behavioural disorders may complicate the surgical treatment of patients.

**Case presentation:**

We present the case of an 11-year-old British Caucasian girl with Smith-Magenis syndrome who developed a severe, progressive thoracic and lumbar scoliosis measuring 85° and 80°, respectively. She had no cardiac or renal anomalies. Brace treatment was unsuccessful to prevent deterioration of the scoliosis. Both curves were rigid on supine maximum side-bending and traction radiographs. Our patient underwent a posterior spinal arthrodesis with pedicle screw/hook and rod instrumentation and autologous iliac crest graft, supplemented by allograft bone. She had an uneventful postoperative course other than the development of a small wound dehiscence which required resuturing with no signs of a wound infection. A good correction of both scoliotic curvatures to 45° and 40° and a balanced spine in both the coronal and sagittal planes was achieved. Follow-up to skeletal maturity (4 years post-surgery) showed no loss of deformity correction, no detected pseudarthrosis and a good clinical outcome.

**Conclusion:**

Patients with Smith-Magenis syndrome can develop a severe scoliosis that may require surgical treatment. Congenital cardiac and renal disease, immunodeficiency and severe behavioural problems can affect the surgical outcome following spinal arthrodesis and need to be taken into consideration. Our case demonstrates that surgical correction of the deformity can be performed safely on this group of patients, with a good outcome and an uncomplicated postoperative course.

## Introduction

Smith-Magenis syndrome is a rare syndromic condition associated with an interstitial deletion of the short arm of chromosome 17 (17p11.2) containing the retinoic acid-induced 1 (RAI1) gene or due to mutation of RAI1. The genotype was first reported by Smith *et al. *[1], in two patients with cleft palate and congenital heart disease. Subsequently, a characteristic phenotype which includes craniofacial, musculoskeletal, neurological, behavioural and systemic abnormalities has been described [2,3]. The clinical features of the Smith-Magenis phenotype can be easier recognised in late childhood. However, babies and young children often present with global developmental delay and only mild dysmorphic features, making a clinical diagnosis difficult. In this case, the diagnosis is established through genetic testing using fluorescence *in situ *hybridisation (FISH), while multiplex ligation-dependent probe amplification (MLPA) and real-time quantitative PCR are the newer, cost-effective, and high-throughput technologies [2,4].

Craniofacial features of the condition include midface hypoplasia, brachycephaly, a broad face with a wide nasal bridge, a down-turned upper lip, synophrys, frontal bossing, prognathia, as well as abnormal ears with associated hearing impairment. Ocular characteristics include iris abnormalities, microcornea, myopia, strabismus, and retinal detachment. Laryngeal anomalies producing a hoarse voice are also common.

Musculoskeletal features include a short stature, short broad hands (particularly brachydactyly affecting the ring and little fingers), syndactyly, pes planus or pes cavus, and scoliosis (in at least 30% of patients) [5].

Neurological problems include epilepsy, hypotonia, peripheral neuropathy, global developmental delay (affecting primarily the speech and language and secondarily the motor development), and cognitive disorders with an IQ score within the range of 20-78 [6]. Abnormal nerve conduction studies have been reported in a small number of patients [2,6]. Behavioural issues include hyperactivity, a self-mutilating and self-injurious attitude (manifested by head banging, wrist biting, and onychotillomania), temper tantrums, sleep disorders, and self-hugging.

Cardiac abnormalities include atrial and ventricular septal defects, tricuspid and mitral valve stenosis or regurgitation, mitral valve prolapse, subvalvular pulmonary stenosis, and tetralogy of Fallot [6,7]. Renal anomalies include duplication of the collecting system, unilateral renal agenesis, ectopic kidneys, and bladder distension [6].

Endocrine abnormalities include thyroid dysfunction producing thyroxine deficiency and low immunoglobulin levels resulting in susceptibility to infection.

In a recent study, Edelman *et al*. [8] demonstrated a broad range of clinical features and variable levels of involvement in Smith-Magenis syndrome, as well as genotype-phenotype relationships with differences between patients with 17p11.2 deletions and RAI1 mutations. Patients with smaller deletions may have less severe phenotype with a lower chance of seizures and oral-motor complications (breathing, eating, and speaking) compared to patients with common deletions. The specific type of small deletion may also correlate with a lower prevalence of dental anomalies, brachycephaly, iris abnormalities, head-banging, and hyperactivity.

We present a patient with Smith-Magenis syndrome who developed a severe thoracic and lumbar scoliosis and underwent a posterior spinal arthrodesis. We describe the patient's postoperative course and final surgical outcome at skeletal maturity, four years following scoliosis surgery. To the authors' knowledge, this patient constitutes the first detailed report of the clinical course and surgical outcome following spinal deformity surgery in patients with this condition presented in the literature.

## Case presentation

A British Caucasian female patient aged 10 years and 11 months presented to our institution with a thoracic and lumbar scoliosis. She was noted to have the phenotypic features indicative of Smith-Magenis syndrome at the age of three years and the diagnosis was confirmed with chromosomal studies. There was no family history of syndromic conditions or scoliosis.

The development of a scoliosis was first noted at the age of 4 years and the patient was treated with an underarm spinal brace in another spinal centre. The deformity remained stable up to the age of 10 years when the curvature progressed rapidly as the patient started going through her pubertal growth spurt. During that stage, the brace was discontinued as the patient's compliance was very poor and any brace effectiveness was limited due to the fact that the patient was significantly overweight. At presentation to our clinic, she remained premenarchal with a body weight of 63 kg which was above the 95th percentile for sex- and age-matched normal population. Her arm span was 142 cm which was between the 25th and 50th percentiles for stature and her BMI was calculated to be 31.2.

On clinical examination, the patient was found to be very hyperactive. Overall, she had a balanced spine with level shoulders and pelvis despite a mild thoracic translocation to the right and no leg length discrepancy. However, she had a severe thoracic and lumbar deformity with a significant rib hump adjacent to the convexity of the thoracic scoliosis, as well as a marked waist line asymmetry with prominence of the pelvis on the concavity of the lumbar curve. There were no skin or soft tissue abnormalities overlying the spine. The patient was a community ambulator and had no neurological abnormality. Neurological examination showed normal tone, muscle power, sensation and tendon reflexes in the upper and lower limbs, as well as symmetrically elicited abdominal reflexes. There were no upper motor neurone signs.

Radiographs of the spine during the initial assessment in our clinic revealed a right thoracic scoliosis extending from T4 to T10 and measuring 75° and a left lumbar scoliosis extending from T11 to L4 and measuring 65°. Both curvatures were rigid on supine maximum side-bending and traction radiographs. The radiological evaluation excluded the presence of congenital abnormalities in the development of the vertebral column and the chest wall. There were no features suggestive of congenital spinal stenosis and the interpedicular distance was within normal limits. The Risser grade was 0, indicating that the patient had a significant amount of remaining growth and this could result into further deterioration of her scoliosis.

### Preoperative assessment

An MRI of the spine demonstrated no intraspinal anomalies, normal appearance of the pedicles, no evidence of spinal stenosis, and normal kidneys. An ultrasound of the kidneys and the bladder did not elicit pathological findings. A cardiological examination showed normal cardiac function. A respiratory assessment did not identify any abnormality. An anaesthetic evaluation confirmed the fitness of the patient to undergo scoliosis surgery and excluded laryngeal anomalies that could complicate intubation. Blood tests demonstrated lower than normal immunoglobulin levels.

### Spinal procedure

**Figure 1:**
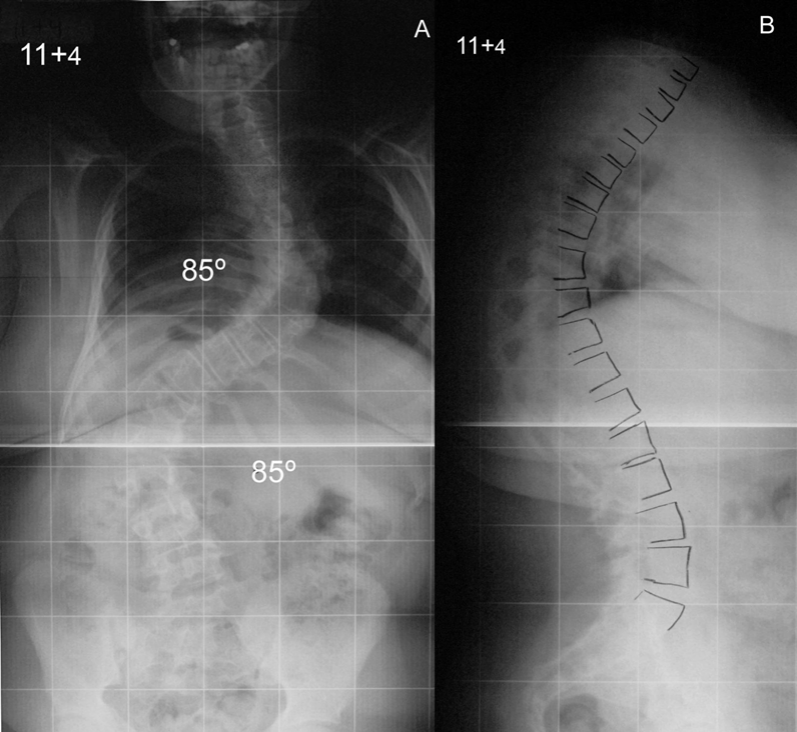
**Preoperative posteroanterior (A) and lateral (B) radiographs of the spine show a thoracic and lumbar scoliosis measuring 85 and 80° respectively with increased thoracic kyphosis**.

Our patient underwent a posterior spinal arthrodesis extending from T3 to L4 with a double pedicle hook/screw and rod construct six months following initial clinical assessment and while the scoliosis had further progressed (Figure 1). An interfacetal and intertransverse arthrodesis was performed bilaterally at every level across the instrumentation using iliac crest graft supplemented by allograft bone. Spinal cord monitoring was performed during the surgery recording somatosensory (SSEP) and motor (MEP) evoked potentials and there were no problems.

Continuous arterial line blood pressure, as well as ECG monitoring was performed throughout the procedure. Central venous access was also placed to allow for rapid administration of fluids and blood products. Intraoperative blood conservation techniques were applied with the use of a cell saver. Three doses of a first generation cephalosporin were administered immediate preoperatively, at completion of surgery and eight hours later as per routine protocol in our institution.

### Postoperative recovery

In order to avoid problems related to patient's hyperactivity, as well as her tendency towards a self-injurious behaviour, the period of sedation and assistive ventilation after scoliosis surgery was electively prolonged. The patient was extubated three days following the procedure. During this initial postoperative period nutrition was maintained with nasogastric feeding. She was fitted with a plastazote spinal brace in order to provide additional support to the spine and limit spinal mobility. Six days following surgery, our patient developed a superficial dehiscence of the distal third of the spinal wound; this was treated with resuturing and healed without further problems. There was no sign of a wound infection and both the blood and wound cultures were negative.

**Figure 2:**
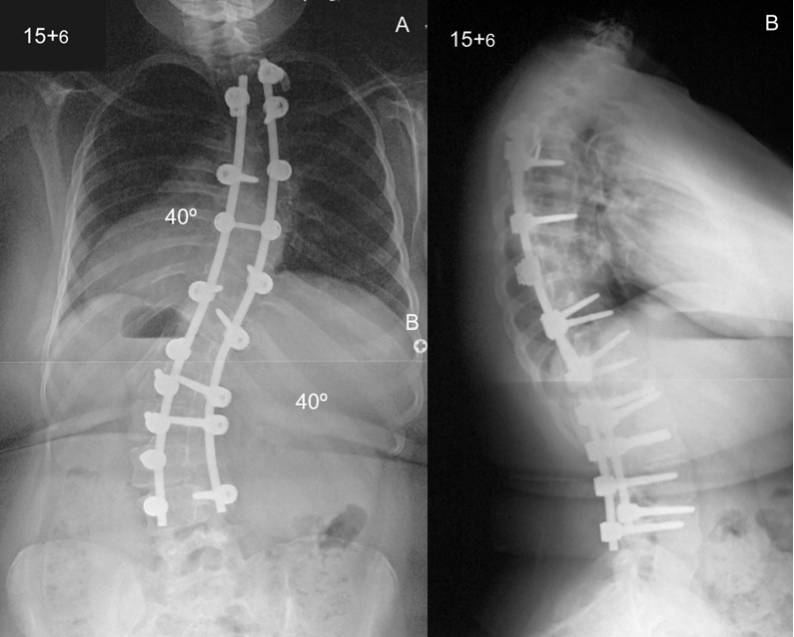
**Postoperative posteroanterior (A) and lateral (B) radiographs of the spine show correction of the thoracic and lumbar scoliosis to 45 and 40° and a balanced spine in both the coronal and sagittal planes**.

Postoperative radiographs showed correction of the thoracic scoliosis from 85° to 45° and the lumbar scoliosis from 80° to 40° with a balanced spine in the coronal and sagittal planes (Figure 2). The patient was discharged 10 days after the initial procedure. At the latest follow-up four years following surgery, the patient had no complaints of her back and had a good level of activities within the limitations of her condition. She was skeletally mature as determined by the Risser grade which was 5. There was no loss of scoliosis correction across the instrumented levels and no detected pseudarthrosis. There was also no evidence of add-on deformity either above or below the levels of the spinal fusion.

## Discussion

Smith-Magenis syndrome has an incidence of approximately 1 in 25,000 live births and is a rare cause of scoliosis [4,8]. Even though the condition is caused by an interstitial deletion within the short arm of chromosome 17 (17p 11.2), there is variability in the size of the deletion and the nature of the genetic change [9]. There is also variability in the expressed phenotype and a poor correlation between genotype and phenotype [10].

The natural history of the scoliosis in association with Smith-Magenis syndrome has not yet been clearly defined. The pattern of spinal deformity is not consistent in previous series and not all reported patients have undergone surgical intervention [5]. In addition, there is no available information on long-term follow-up of patients who underwent scoliosis correction at a young age in order to determine whether there was recurrence or add-on deformity as the consequence of the remaining spinal growth. Moreover, the condition is associated with several features, such as congenital heart disease, a self-destructive behaviour, and immunoglobulin deficiency that could be considered relative contraindications to scoliosis surgery.

Spilsbury *et al*. [5] reported on 22 patients with Smith-Magenis syndrome among whom seven patients developed a scoliosis (30%). The age of the whole group ranged from 4-26 years; 14 patients were female and 8 male. The curve types were described as right thoracic in 3 patients, left thoracolumbar in 3, and double major in one patient. Curve size ranged from 18° to 113°. One patient was reported to have a thoracic lordosis. There was no radiological evidence of congenital vertebral anomalies. One patient had a rapidly progressive curve which deteriorated from 38° to 75° in six months. In all patients the scoliosis was stiff. No neurological abnormalities were found on clinical examination. Three of the seven patients with a scoliosis were treated surgically due to progression of the deformity. One patient underwent a staged anterior release and posterior instrumented fusion and two patients had posterior scoliosis surgery only.

Greenberg *et al*. [6] recorded the development of a mild to moderate scoliosis in 13 out of 20 patients (65%) of at least four years of age with Smith-Magenis syndrome and this was most commonly located in the mid-thoracic region but gave no information on the need for treatment.

Our patient developed a severe thoracic and lumbar scoliosis which progressed during the rapid stages of spinal growth. There was no evidence of congenital vertebral, cardiac or renal anomalies despite the known association of these clinical features with Smith-Magenis syndrome. The patient had no neurological abnormalities and the pattern of her deformity resembled an adolescent idiopathic scoliosis with the response to surgical treatment and postoperative course being very similar. Intra-operative spinal cord monitoring was performed effectively with the use of both SSEP and MEP and they showed no abnormalities throughout the procedure.

The presence of an inherently poor immune system in this group of patients could increase the risk for a postoperative infection and this could significantly compromise the outcome of the scoliosis surgery. Despite a low immunoglobulin count, our patient made an uneventful postoperative recovery and her spinal arthrodesis healed without complications of wound infection or pseudarthrosis. Long-term follow-up to skeletal maturity showed no loss of scoliosis correction, a balanced spine and a good functional outcome.

The postoperative course in patients with Smith-Magenis syndrome can also be challenging due to the significant behavioural disorders that include an aggressive, maladaptive and self-injurious behaviour [11]. Our patient was electively ventilated following scoliosis surgery and over a period of 3 days and we believe that this allowed for a less disruptive behaviour and a smoother postoperative recovery. The application of a spinal brace for four months post-surgery aimed to provide additional support to the spine and may have reduced the chance of instrumentation failure in the postoperative period in a patient with a high risk of repetitive injuries.

## Conclusion

Scoliosis is a common manifestation of Smith-Magenis syndrome with a reported prevalence of 30-65% [5,6]. Regular screening of this group of patients is recommended so that an early diagnosis can be made and a severe scoliotic deformity may be avoided. If a progressive curvature develops in a young child, surgical correction of the deformity is likely to be required. Our report has demonstrated that scoliosis correction can be achieved safely with no increased risk of intra- or postoperative complications. A meticulous multidisciplinary pre-operative assessment will detect any associated comorbidities and reduce the potential risk for severe complications. Nursing of patients with this condition following spinal arthrodesis can be challenging due to their behavioural issues and will require close cooperation of the medical and nursing teams with the family of the patient. However, a good surgical outcome can be achieved and maintained at follow-up.

## Abbreviations

BMI: body mass index; ECG: electrocardiogram.

## Consent

Written informed consent was obtained from the patient's next-of-kin for publication of this case report and any accompanying images. A copy of the written consent is available for review by the Editor-in-Chief of this journal.

## Competing interests

The authors declare that they have no competing interests.

## Authors' contributions

AT was involved in the conception and design, analysis of the data, preparation of the manuscript, final approval of the version to be published. AB did the data analysis, and the preparation of the manuscript. CM was involved in the collection of data, and participated in writing the initial version of the manuscript. All authors have read and approved the final manuscript.
